# Restorative Treatment of Amelogenesis Imperfecta with Prefabricated Composite Veneers

**DOI:** 10.1155/2021/3192882

**Published:** 2021-08-02

**Authors:** Claudio Novelli, Maurizio Pascadopoli, Andrea Scribante

**Affiliations:** ^1^DENS Centro Medico Lombardo, Milan 20124, Italy; ^2^Unit of Orthodontics and Pediatric Dentistry, Section of Dentistry, Department of Clinical, Surgical, Diagnostic and Pediatric Sciences, University of Pavia, 27100 Pavia, Italy

## Abstract

This case report presents the use of prefabricated composite veneers for restorative treatment of amelogenesis imperfecta (AI). This technique bridges the gap between a conventional direct technique and a conventional indirect technique and introduces an alternative semidirect restorative technique for AI patients. The aim of this case report is to describe restoration of a young girl with severe AI using prefabricated composite veneers and to discuss the benefits and limitations of this technique compared to the alternative restorative techniques.

## 1. Introduction

Amelogenesis imperfecta (AI) is a genetic disease caused by mutation of the genes involved in the presecretory, secretory, and maturation stages of enamel formation [1–4]. The mutation can affect both the primary and permanent dentitions and can be passed on from parents to children or develop in individuals with no family history [5, 6]. Depending on the gene involved and the timing when the disruption occurs, AI produces a wide range of enamel alterations ranging from superficial discoloration to complete enamel agenesia. Based on the phenotype of the enamel alteration, AI is classified as type I hypoplastic, type II hypomatured, type III hypocalcified, and type IV hypomatured-hypoplastic. When not only the enamel phenotype but also the inheritance pattern is considered, fifteen AI subtypes are classified. These fifteen subtypes are currently the most widely accepted AI classification system (Table 1) [7–9].

Although AI is primarily an enamel disease, not enamel-related disorders are frequently reported such as pulpal calcifications, delayed tooth eruption, congenital missing teeth, root resorption, open bite, negative overjet, and altered vertical jaw relationship [10–13]. On account of the diverse clinical manifestations, the successful treatment of AI patients requires a multidisciplinary team including a pediatric dentist, a restorative dentist, a prosthodontist, an orthodontist, and a maxillofacial surgeon [14]. Treatment always starts with restoration of the involved dentition, and minimally invasive restoration with direct composite is highly recommended due to the young age of many AI patients. However, a direct composite does not perform well in AI patients and clinical studies reveal limited longevity and high failure rate especially in AI type II and type III where the enamel qualitative alterations produce suboptimal etching pattern and impaired bond strength [15–17]. Enamel deproteinization with NaOCl was suggested to improve bond strength in type II AI patients with hypocalcified enamel and increased protein content [18, 19]. However, very limited in vivo studies are available to support the technique and the results of such studies are not always conclusive [20].

In a recent retrospective clinical study of 326 composite restorations in AI patients, the failure rate was 2.5 times higher than the control group with debonding, fracture, and secondary decay being the most frequent reasons for failure. The longevity of the composite restorations was shorter, and their quality was poorer in patients with hypomineralised/hypomaturated AI than in patients with hypoplastic AI [21]. Because of these limitations, ceramic crowns are often recommended as an alternative to direct composite especially for treatment of severe AI and multisurfaced lesions. Ceramic crowns provide excellent esthetic and function in AI patients with a longevity similar to unaffected patients and no correlation between clinical performance and type of enamel alteration [21–23]. In a split mouth study on the long-term outcome of 227 crowns in AI patients, Lundgren et al. reported a survival rate of 99.6% after 5 years with no significant difference between crowns fabricated with Procera (veneered zirconia ceramic) and IPS e.max (lithium disilicate ceramic) [24]. However, ceramic crowns involve a significant sacrifice of tooth structure and a high risk of pulp exposure in young patients with prominent pulp horns. Ceramic crowns in young patients also involve high risk of esthetic failure due to exposed margins of the restoration following craniofacial growth and soft tissue maturation [25–27].

Unfortunately, many AI patients fall in the young age group where ceramic crowns are not indicated and direct composite restorations do not perform well. When neither indirect ceramics nor direct composite seem to be the right answer for the AI patient, this paper presents an alternative restorative technique with prefabricated composite veneers. This technique bridges the gap between the conventional direct composite and the indirect ceramic techniques [28] and is indicated for the treatment of young AI patients because it provides an esthetic and functional restoration in a single appointment with minimal sacrifice of the tooth structure.

The aim of this case report is to describe the restorative treatment of a young patient with severe AI using prefabricated composite veneers and to discuss the benefits and limitations of this technique compared to the alternative restorative techniques.

## 2. Case Report

The patient was a 9-year-old girl with a family history of AI. At the time of the visit, the patient was in a stage of mixed dentition but all the primary teeth had been extracted by her primary dental care provider due to multiple secondary decays. The permanent first molars and the upper central incisors were less severely involved and successfully restored with direct composite.

The patient was referred for treatment of the lower incisors very sensitive upon thermal stress and unsightly due to abnormal shape, size, and color. The appearance of the lower incisors made the girl uncomfortable with her smile, and she reported bullying at school because of her teeth.

**Figure 1:**
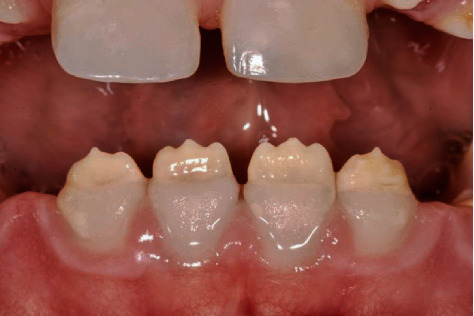
Preoperative view of the lower incisors affected by AI type l.

**Figure 2:**
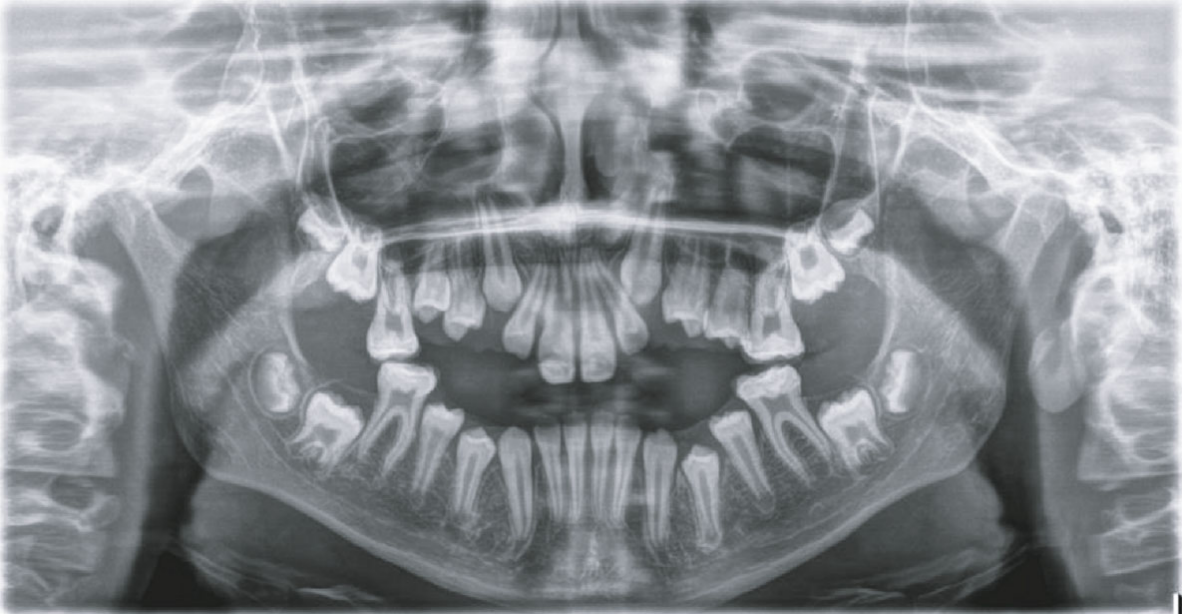
Preoperative panoramic radiographic view.

Intraoral clinical examination of the lower incisors revealed missing enamel in the incisal half and reduced enamel thickness in the cervical half of the teeth (Figure 1). Radiographic examination showed normal enamel radiopacity and normal contrast with the underlying dentin (Figure 2).

Based on the clinical and radiological examinations, a diagnosis was made of AI type I according to the Witkop classification [7]. Type l is the most common form of AI caused by a mutation in the enamelin gene ENAM 4q215 transmitted by autosomal dominant inheritance. This mutation introduces a disruption in the presecretory and secretory stages of amelogenesis resulting in a layer of hypoplastic enamel with normal mineralization but reduced thickness [28–30].

The treatment options for restoration of the lower incisors were discussed with the patient and her parents, and a final decision was made to restore the teeth with prefabricated composite veneers.

Once the treatment plan with prefabricated composite veneers was approved, the clinical procedure started with veneer size selection.

**Figure 3:**
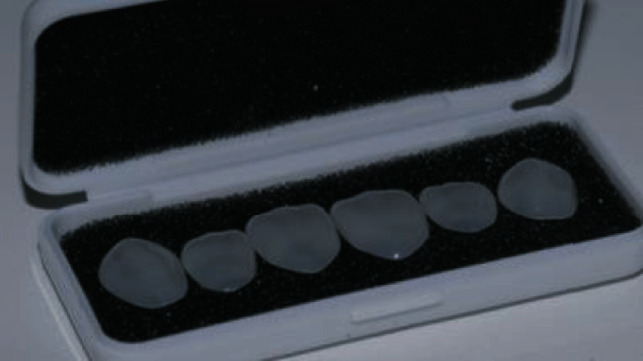
Edelweiss prefabricated composite veneers.

**Figure 4:**
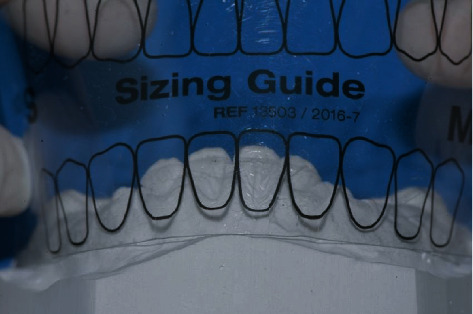
Edelweiss Veneer size selection using the Edelweiss sizing guide.

The prefabricated composite veneers for the patient in this report (Edelweiss Veneers, Edelweiss Dentistry, Wolfurt, Austria) (Figure 3) are available in four sizes (XS, S, M, and L) based on average tooth dimensions in the human population. A custom sizing guide is included in the system to select the veneer that best fits the patient (Figure 4).

**Figure 5:**
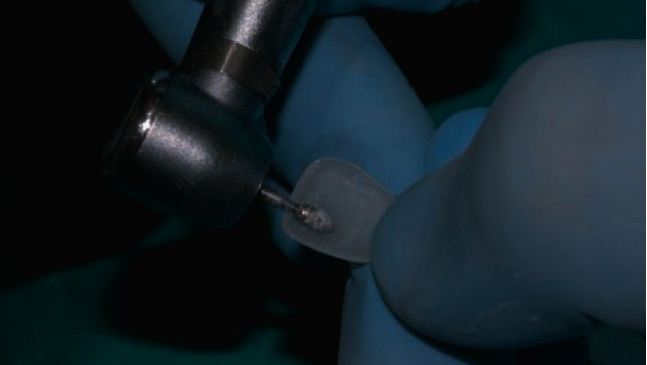
Internal adjustments to reduce the thickness of the veneer.

**Figure 6:**
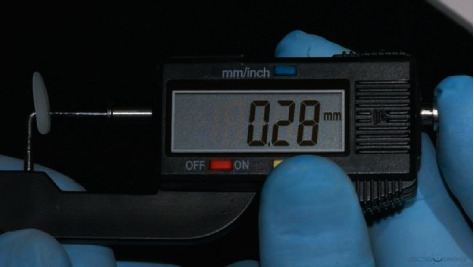
Final veneer thickness measured with a digital caliper.

If none of the available sizes fits the patient's teeth, the width and length of Edelweiss Veneers can be altered to accommodate specific dimensional requirements. For the patient in this report, Edelweiss Veneer size M was selected, and no width or length alteration was required. However, the thickness of the veneer was reduced with a football shape diamond bur (8379, Komet USA, Rock Hill, SC, USA) to allow a more conservative tooth preparation (Figures 5 and 6). Using the same football shape diamond bur, the cervical margin of the veneer was finished to a knife-edge configuration for maximum tissue preservation in the gingival area where the enamel layer is thinner. Thanks to the combination of minimal thickness of the composite laminate and knife-edged configuration of the cervical margin, no tooth preparation was required and the thin hypoplastic enamel layer of the AI patient was fully preserved.

**Figure 7:**
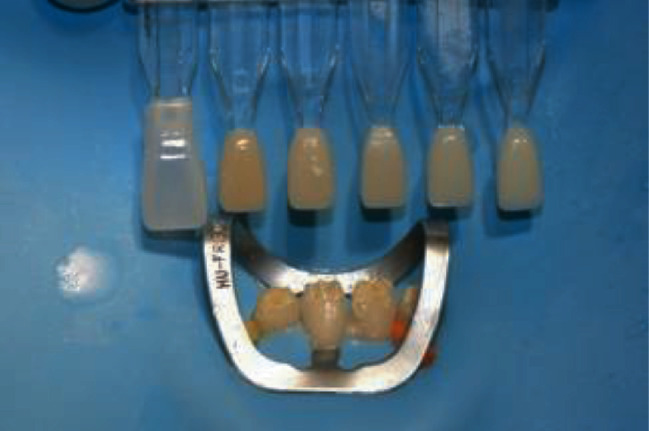
Shade selection of the luting composite using Edelweiss custom shade guide.

After veneer size selection, the next step was choosing the shade of the luting composite (Figure 7). Luting composite color selection is a critical step for successful restoration with Edelweiss Veneer because the laminate is fabricated with a colorless enamel shade, and the final color of the veneer is determined by the color of the luting composite. The Edelweiss Veneer System includes a high-viscosity nanohybrid composite for cementation available in several dentin and enamel shades (Edelweiss NH, Edelweiss Dentistry, Wolfurt, Austria). For the patient in this report, Edelweiss NH shade A2 was selected with the addition of an opalescent flowable composite in the incisal area (Effect Blue, Edelweiss Dentistry, Wolfurt, Austria) to increase incisal translucency and highlight the halo effect.

**Figure 8:**
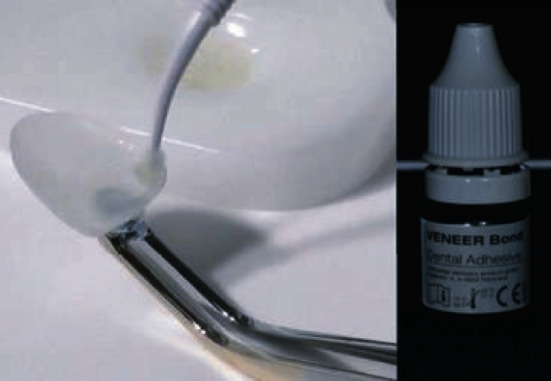
Veneer Bond application.

After size and shade selection, the intaglio of the veneer was conditioned with a proprietary resin primer (Veneer Bond, Edelweiss Dentistry, Wolfurt; Austria) applied with a microbrush and light cured 20 seconds according to the manufacturer recommendations (Figure 8). No acid-etching, no sandblasting, and no silane application are required inside Edelweiss Veneer. However, the manufacturer recommends internal conditioning with Veneer Bond to promote chemical adhesion and to increase bond strength between the highly inorganic laminate and the luting composite.

**Figure 9:**
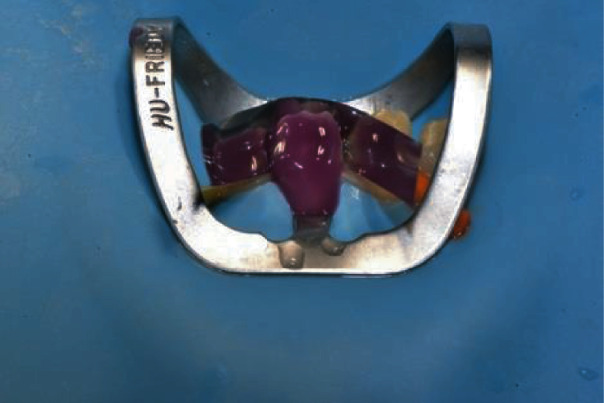
Tooth etching 35% H_3_PO_4_.

**Figure 10:**
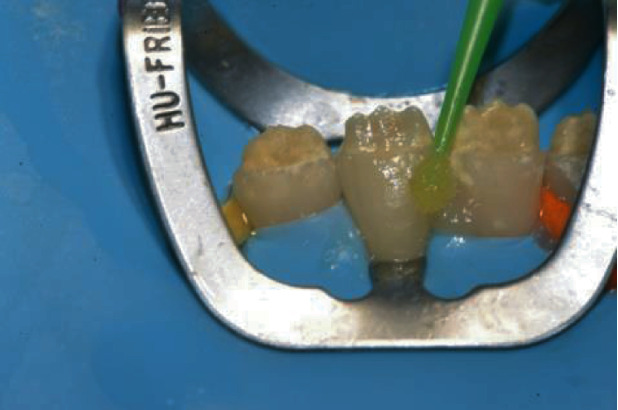
Application of single-component adhesive.

After Veneer Bond application, the veneers were ready for delivery and the working field was isolated with a rubber dam and a 212 Hu-Friedy clamp and the first lower incisor was etched with 37% H3PO4 (Gel Etchant, KerrHawe, Bioggio, Switzerland). Etching gel application started from enamel and after 15 seconds moved to dentin for another 15 seconds for 30 s (Figure 9) followed by water rinsing for 30 s and application of a single-step adhesive according to the manufacturer instructions (Scotchbond Universal, 3 M ESPE, Seefeld, Germany) (Figure 10).

**Figure 11:**
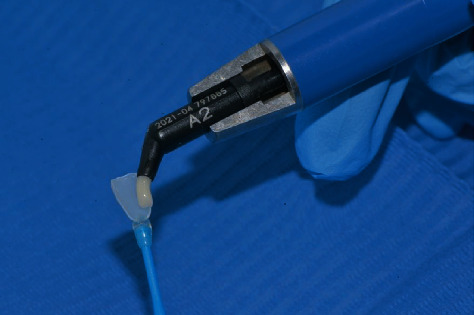
Veneer loaded with the selected composite shade.

**Figure 12:**
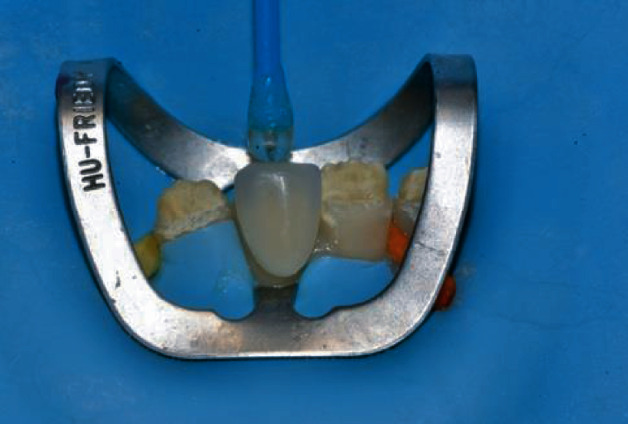
Veneer seated on the deserving tooth.

Then, the veneer was loaded with the selected composite shade (Figure 11) and seated on the deserving tooth (Figure 12).

**Figure 13:**
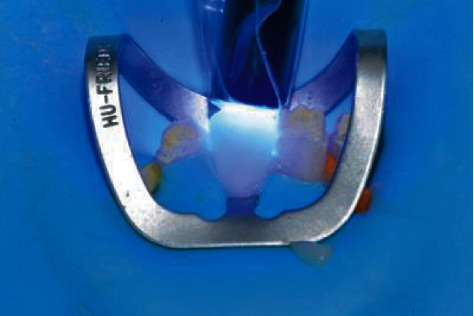
Veneer light cured on the tooth.

After gently pressing the veneer in position, the extra composite was removed with a thin spatula (CompoSculp DD 9/10, Hu-Friedy, Chicago, Ill, USA) and carefully sculpted to achieve optimal adaptation between the veneer and the tooth. Then, the veneer was light cured 20 seconds from the lingual and 20 seconds from the buccal using a high-power (1.330 mW/cm2) curing light (Demi Plus, Kerr Corporation, Brea, CA USA) (Figure 13).

**Figure 14:**
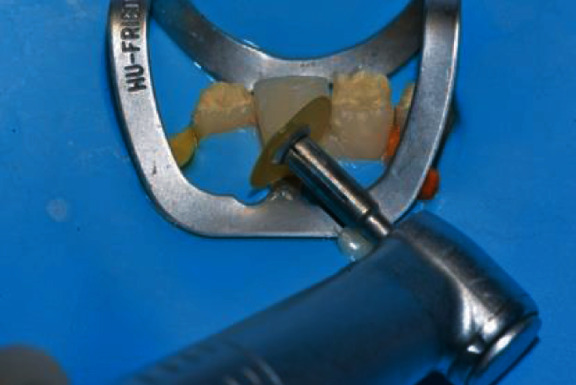
Finishing the margins with composite finishing discs.

**Figure 15:**
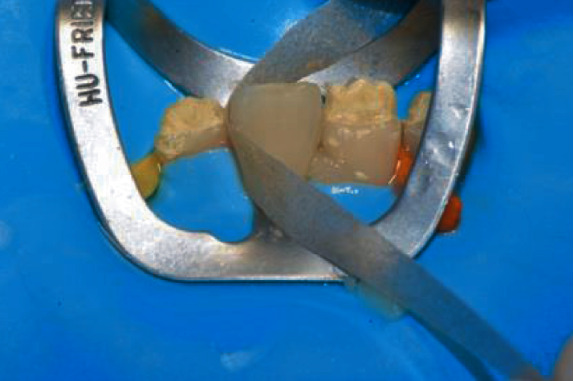
Interproximal finishing with composite paper finishing strip.

Finally, the margins of the veneer were finished with composite finishing discs (Sof-Lex XT, 3M ESPE, Seefeld, Germany) (Figure 14) and interproximal finishing strips (Sof-Lex, 3M ESPE, Seefeld, Germany) (Figure 15) followed by a diamond-impregnated silicone cup (Dia step 2, Ravelli, Milano, Italy) at 7500–10,000 rpm under water to produce the final luster.

**Figure 16:**
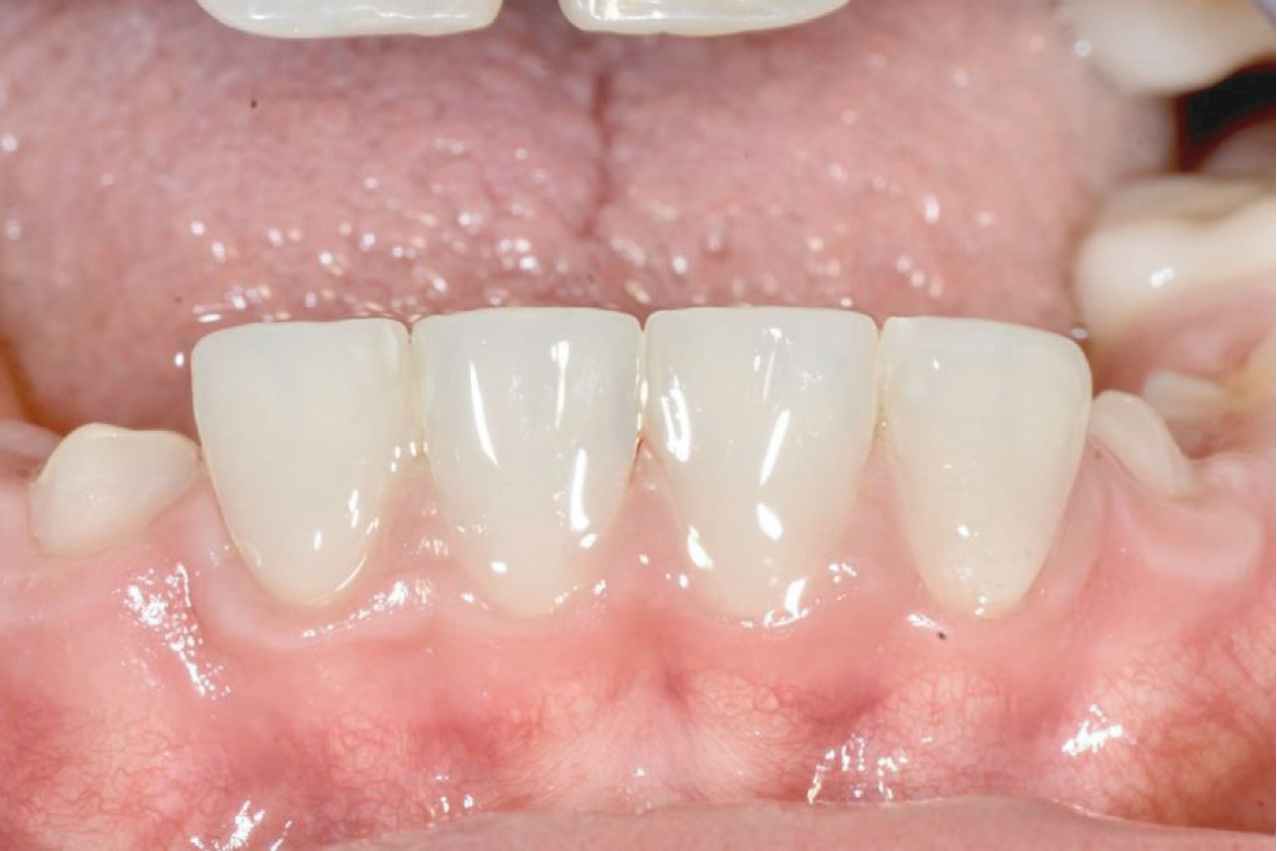
2 weeks postoperative view of Edelweiss Veneers [31–41].

**Figure 17:**
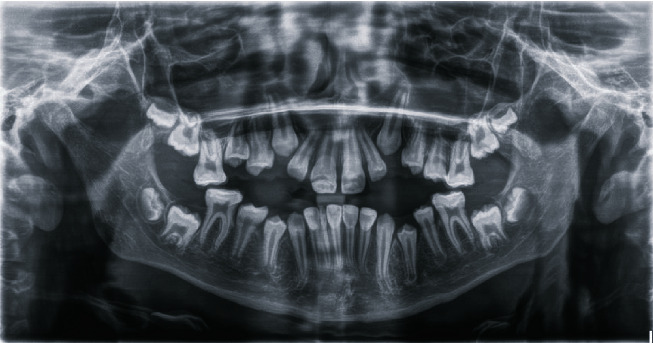
Postoperative panoramic radiographic view.

Once the same step by step clinical procedure was completed for all the four veneers, the patient was dismissed and rescheduled for postoperative evaluation after two weeks. At the recall appointment functional evaluation (absence of fractures, marginal adaptation), biological evaluation (soft tissue response, postoperative sensitivity), and esthetic evaluation (gloss, color matching) were completed and resulted fully satisfactory (Figure 16). Radiological examination showed successful integration of the restorations (Figure 17). The patient was happy with the esthetic outcome and reported that hypersensitivity disappeared after placing the veneer.

**Figure 18:**
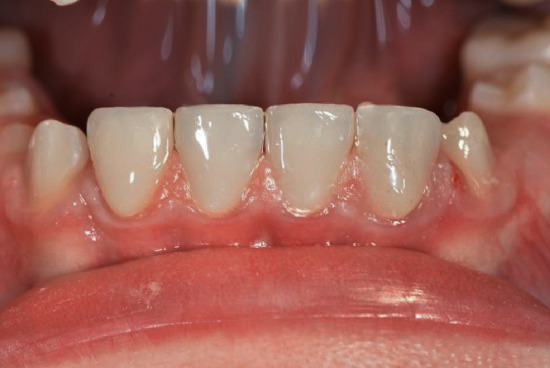
6 months postoperative view of Edelweiss Veneers [31–41].

A second recall appointment was scheduled 6 months after delivering the veneers. At the new follow-up visit, the veneers resulted fully functional with no marginal discoloration and no alteration of the original superficial luster (Figure 18). The veneers showed good soft tissue response, and the patient's oral hygiene was significantly improved as reported by the RDH who has been following the patient since the initial phase of the treatment. AI patients often experience difficulty in maintaining good oral hygiene on account of the increased tooth sensitivity that makes tooth brushing uncomfortable and the rough tooth surface that facilitates plaque accumulation. Also the impaired smile appearance with abnormal tooth shape, size, and color contributes to poor oral hygiene motivation [31–33]. Previous research demonstrates that a strong correlation exists between attractive smile appearance and positive oral health behaviors [34], and a secondary benefit of the esthetic restoration of the young AI patient presented in this report was the positive impact on the patient's strive to maintain optimal oral health.

## 3. Discussion

The currently available literature recommends using either direct or indirect techniques for restoration of AI patients and indicates the age of the patient as well as the extension of the enamel lesions as the main decisional criteria [31]. However, other factors should be included in the decision-making process such as the degree of esthetic alteration and the patient's esthetic expectation. AI is often associated with alterations of tooth color, size, and shape that compromise the natural appearance of the smile and have a negative impact on the oral health-related quality of life of the patient [32, 34]. AI patients frequently report being teased about their dental appearance and develop higher levels of social avoidance and psychological distress compared to the unaffected population [35, 36]. This effect is age-dependent, and young AI patients show more psychological disturbance than adult AI patients [37–39]. Hence, esthetic restoration is critical for the successful treatment of young AI patients where the smile should be restored to natural and healthy appearance despite the challenges associated with the young age. A major challenge is the limited compliance on the dental chair that impairs optimal implementation of conventional restorative techniques involving multiple clinical steps and extended chair time. A benefit of the prefabricated composite veneer technique is the simplified clinical procedure that produces a restoration with ideal shape, accurate anatomy, and glossy surface in a single appointment with a limited number of clinical steps and reduced chair time. In the case presented in this report, the clinical procedure was further expedited by preparing the veneers in advance on the study model (select size, adjust thickness, etc.) thereby reducing the actual chair time for each veneer to approximately 10 minutes, a time frame that every young patient can easily cope with.

Another benefit of the prefabricated composite veneer technique is the reduced thickness of the laminate that allows minimally invasive tooth preparation and maximum enamel preservation. Maximum enamel preservation is mandatory for AI type I patients where the thickness of the hypoplastic enamel layer is reduced and conventional tooth preparation easily leads to dentin exposure. Even if the initial preparation is intraenamel, after one or two cycles of veneer replacement, the thin enamel layer is likely to be lost thus limiting the option of a new veneer and leading to more invasive full coverage restoration and additional tissue loss. A secondary benefit of the reduced thickness of the prefabricated composite veneer is the optimal contact lens effect and invisible margin in the gingival area where the laminate tapers to zero. The invisible margin is a critical factor for successful restorative treatment in young patients because it reduces the risk of esthetic failure due to margin exposure following craniofacial growth and soft tissue maturation. A small margin exposure is unlikely to be an issue because the margin of the prefabricated composite veneer is not visible while a larger margin exposure can be conveniently repaired intraorally with the addition of direct composite. Intraoral composite repair increases the longevity of the prefabricated composite veneer because it allows minimally invasive intervention as opposed to replacement of the restoration with many biological and financial benefits for the patient [40–44].

The successful esthetic outcome, the simplified clinical procedure, the minimally invasive tooth preparation, the convenient intraoral repair, and the possible bonding of orthodontic brackets for orthodontic treatment to be carried out in the future were the main benefits of the prefabricated composite veneer technique for the AI patient presented in this report. Nonetheless, the technique showed some limitations such as the higher cost compared to the conventional direct composite technique. Even if the extra cost of the prefabricated composite veneer is partially compensated by the shorter chair time, the treatment fee for the patient is higher especially when multiple veneers are needed. Another limitation is the limited data available regarding the performance of prefabricated composite veneers compared to the alternative anterior restorations (i.e., direct composite and indirect ceramics). In laboratory studies, prefabricated composite veneers perform better than direct composite veneers because the extraoral curing process under controlled temperature, light, and pressure produces a significant improvement in color stability, superficial roughness, and microhardness [45–47]. The Edelweiss Veneers used for the patient in this report are expected to perform even better because the addition of high pressure (100 Pa) and high temperature (300°C) to the curing process further improves mechanical and physical properties of the composite laminate [48]. However, no clinical studies have been published yet and it is not known how the clinical performance and longevity of prefabricated composite veneers compare within direct ceramics that are considered the gold standard for anterior restoration. It is possible that indirect ceramics provide better clinical performance and durability on account of ceramic superior mechanical properties and resistance to aging. However, indirect ceramics have limited application for restoration of young AI patients (like the girl presented in this report) because ceramic crowns involve a high risk of pulp exposure [49–51] and ceramic veneers are likely to be negatively affected by the enamel quantitative and qualitative alterations [52, 53]. When the indirect ceramic restorations are not indicated, prefabricated composite veneers provide an alternative technique for an esthetic and functional restoration of young AI patients that allows to produce a restoration with proper shape, accurate anatomy, and glossy surface with minimally invasive tooth preparation.

## 4. Conclusions

The treatment of severely compromised teeth in young patients suffering from amelogenesis imperfecta can be a challenge for the dental clinician, considering the kind of the disorder and the commonly known low compliance of children. The use of prefabricated veneers can be a valid approach because of the minimally invasive procedures involved and the higher esthetic result obtained, actually introducing an alternative “semidirect” restorative technique.

## Figures and Tables

**Table 1:** Witkop classification of amelogenesis imperfecta 1989 [7].

Type	I	Hypoplastic

	IA	Hypoplastic, pitted autosomal dominant
	IB	Hypoplastic, local autosomal dominant
	IC	Hypoplastic, local autosomal recessive
	ID	Hypoplastic, smooth autosomal dominant
	IE	Hypoplastic, smooth X-linked dominant
	IF	Hypoplastic, rough autosomal dominant
	IG	Enamel agenesis, autosomal recessive

Type	II	Hypomaturation

	IIA	Hypomaturation, pigmented autosomal recessive
	IIB	Hypomaturation, X-linked recessive
	IIC	Snow-capped teeth, X-linked
	IID	Snow-capped teeth, autosomal dominant

Type	III	Hypocalcified

	IIIA	Autosomal dominant
	IIIB	Autosomal recessive

Type	IV	Hypomaturation-hypoplastic with taurodontism

	IVA	Hypomaturation-hypoplastic with taurodontism, autosomal dominant
	IVB	Hypoplastic-hypomaturation with taurodontism, autosomal recessive

## Data Availability

All data are available upon request to the corresponding author.
